# Role of Domiciliary Noninvasive Ventilation in Chronic Obstructive Pulmonary Disease Patients Requiring Repeated Admissions with Acute Type II Respiratory Failure: A Prospective Cohort Study

**DOI:** 10.5005/jp-journals-10071-23115

**Published:** 2019-01

**Authors:** Killen HB Claudett, Michelle Grunauer

**Affiliations:** 1 Universidad de Guayaquil. Facultad de Ciencias Médicas. Guayaquil, Ecuador, Centro Fisiológico-Respiratorio Briones-Claudett, Intensive Care Unit, Panamerican Clinic, Guayaquil, Ecuador, Intensive Care Unit, Ecuadorian Institute of Social Security (IESS), Babahoyo, Ecuador; 2 School of Medicine, Universidad San Francisco de Quito, Quito, Ecuador

## Abstract

**How to cite this article:** Claudett KHB, Grunauer M. Role of Domiciliary Noninvasive Ventilation in Chronic Obstructive Pulmonary Disease Patients Requiring Repeated Admissions with Acute Type II Respiratory Failure: A Prospective Cohort Study. Indian Journal of Critical Care Medicine, January 2019;23(1): 56-57.

Authors in this article evaluate the effectiveness of long-term noninvasive ventilation (LTNIV) in COPD patients requiring frequent hospital admissions and NIV support for acute hypercapnic respiratory failure (AHRF). The authors designed a prospective study and enrolled 120 patients with a history of ≥3 similar episodes in the past year and that survived an admission. Patients received LTNIV (30) or (90) standard treatment alone. Both groups were followed for 1 year.^1^

The authors report significant reductions in mortality, hospital admissions number, ventilator requirements and AHRF and improvement in partial arterial CO2 pressure and severe respiratory insufficiency score in favor of LTNIV group, without significant changes in lung function and exercise tolerance.

We have some remarks on this interesting study:

## First

Patients were instructed to continue with their home NIV immediately after discharge from the hospital as a direct continuation of acute treatment.

Relapses of acute hypercapnic respiratory failure (AHRF) can occur in the first 48 hours of cessation of NIV and on the other hand, there is evidence that patients can become spontaneously eucapnic and thus form part of the patients that do not need long-term NIV at home.^2^

## Second

The authors described a mean positive inspiratory airway pressure of 15.4 cm H2O (range 12 to 18 cm H2O) and the mean positive expiratory airway pressure (EPAP) was 7.4 cm H2O.

Studies carried out in patients with chronic hypercapnic respiratory failure (AHRF) have shown that greater Inspiratory pressures are necessary to improve gas exchange.

In randomized and controlled studies, high-intensity NIV has been beneficial in 1-year mortality in populations with such characteristics.^3^

## Third

The authors do not provide clear definitions regarding the existence of associated co-morbidities such as obstructive sleep apnea, obesity hypoventilation syndrome, heart failure, etc. This raises the question of whether the presence of an underlying condition in both groups could have influenced the results. Data such as body mass index, cardiac arrhythmia, heart failure, or assessment of factors associated with possible obstructive sleep apnea such as (neck circumference, the Epworth sleepiness scale, Mallampati score, etc.) were provided in this study.^4^ With this in mind, this study could include a selected group of patients compliant with NIV which could have original disadvantages at the moment of the interpretation of the primary end result of the study (mortality).
